# Color‐Tunable Organic Nano‐Dots: Synthesis and Applications in Color Conversion and Security Inks

**DOI:** 10.1002/smll.202505043

**Published:** 2025-07-10

**Authors:** Yeasin Khan, Rasheeda Ansari, Khandoker Asiqur Rahaman, Bright Walker, Jang Hyuk Kwon

**Affiliations:** ^1^ Department of Chemistry Kyung Hee University 26 Kyungheedaero, Dongdaemun‐gu Seoul 02447 Republic of Korea; ^2^ Organic Optoelectronic Device Lab. (OODL) Department of Information Display Kyung Hee University 26, Kyungheedae‐ro, Dongdaemun‐gu Seoul 02447 Republic of Korea; ^3^ Center for Biomaterials Biomedical Research Division Korea Institute of Science and Technology Seoul 02792 Republic of Korea

**Keywords:** aqueous dispersion, color conversion layer, color tunable nanodots, energy transfer, FRET, security ink

## Abstract

This study explores the synthesis of water‐based color‐tunable organic nanodots (CTONDs) capable of emitting multiple colors, including white light, by adjusting the molar ratio of blue, green, and red emissive fluorophores in the particles. Spectroscopic analyses reveal that the emissions are due to Forster resonance energy transfer (FRET) between the energy donor and acceptor nanoparticles. The energy transfer efficiencies are high, reaching over 90% in the film state due to the close packing of NDs while in their film state. Various molar ratios produced different colors in both liquid dispersions and in the solid state. These CTONDs demonstrate over 60% color conversion efficiency (CCE) when applied as color conversion layers (CCLs) in light‐emitting devices, maintaining photostability for over four months under ambient conditions. Additionally, their aqueous processability and multicolor tunability make them attractive for environmentally friendly display technologies, flexible optoelectronics, and anti‐counterfeiting applications such as security inks. This work offers a scalable and sustainable approach to fabricating tunable, solution‐processed fluorescent organic nanomaterials and underscores their promise as a versatile platform for next‐generation photonic and optoelectronic applications.

## Graphical abstract

Color‐tunable organic nanodots prepared via micelle‐assisted self‐assembly exhibit efficient Förster energy transfer and multicolor emission, including white light. These aqueous nanodots demonstrate high color conversion efficiency, long‐term stability, and compatibility with solid films and paper substrates. Their tunability, processability, and performance make them promising candidates for eco‐friendly display technologies and anticounterfeiting fluorescent inks.

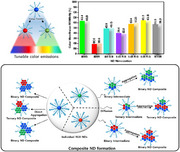

## 1 Introduction

CTONDs have great potential as a focal point of scientific interest, capturing attention due to their remarkable capacity to dynamically alter optical properties in response to external stimuli.^[^ ^1^ ^]^ Their unique attributes, including flexibility,^[^ ^2^ ^]^ lightweight design,^[^ ^3^ ^]^ low power consumption,^[^ ^4^ ^]^ and the ability to produce vivid and customizable colors,^[^ ^5^, ^6^, ^7^ ^]^ position them as versatile candidates for critical roles not only as CCLs for lighting and display applications but also for a wide range of applications where tunable light emission is desired. These roles span an array of fields beyond light‐emitting devices,^[^ ^8^, ^9^ ^]^ such as optical sensors,^[^ ^10^, ^11^ ^]^ security inks,^[^ ^12^, ^13^, ^14^ ^]^ and cutting‐edge lighting solutions. A quantitative understanding of energy transfer efficiency between different organic fluorophore pairs and the ability to accurately control their light emission is a pivotal challenge for unlocking the full potential of these nanodots, facilitating the generation of diverse and vibrant fluorescent emissions. To streamline our discussions, we will denote these pairs as the energy donors and energy acceptors. The energy donor absorbs energy from photons and subsequently transfers this energy to the acceptor moieties. Many recent studies exploring the design of new fluorophores have focused on covalently bonded donor and acceptor moieties for achieving variable colored light emission,^[^ ^15^, ^16^ ^]^ these efforts have grappled with synthetic challenges and struggled to precisely achieve desirable and tunable optical properties such as emission wavelength, full‐width at half maximum (FWHM) and photoluminescence quantum yield (PLQY) necessary for accurate color generation. As a result, there is a growing need for a streamlined and efficient approach to generating light with precise spectral characteristics. Leveraging the structural integrity conferred by the surrounding ligands of organic fluorescent nanomaterials opens avenues for spatial organization and specific binding sites, thereby enhancing the overall efficiency of energy transfer.^[^ ^17^, ^18^, ^19^ ^]^ The added flexibility to vary the ratio between the energy donors and acceptors further enables the fine‐tuning of emission spectra to meet specific application requirements.

Building upon the foundation of our prior study,^[^ ^20^ ^]^ wherein we demonstrated an efficient method for the preparation of aqueous dispersions of fluorescent organic nanoparticles, or “nanodots” (NDs) using nonionic/anionic surfactants to mitigate aggregation quenching and bolster stability, our present approach builds on the strengths of our previous ND approach. Here, we explore the use of non‐covalently bonded energy donor and energy acceptor moieties within the micelle structure of the surfactant, resulting in the creation of micelle‐based fluorescent color‐tunable organic nanodots. This sophisticated assembly exhibits soft, surfactant‐stabilized, organic emissive nanostructures with excellent FRET outputs.^[^ ^21^, ^22^ ^]^ With the added advantage of an easily adjustable molar ratio between the energy donor and energy acceptor moieties, facilitating a wide gamut of color generations alongside white‐light emission.

We've also explored commercially important practical applications of the CTOND system as color conversion layers. Their performance excels compared to state‐of‐the‐art technologies employing quantum dots (QDs). Furthermore, their potential as fluorescent inks or security inks has been demonstrated through the staining of NDs on paper. Impressively, the fluorescence endures even after solvent removal, and the emission color remains stable over time. This robust resilience underscores not only the scientific advancements achieved but also the practical viability of these materials in real‐world scenarios, promising innovation in diverse industries and applications.

## 2 Results and Discussion

### 2.1 Preparation of the ND Aqueous Dispersions

**Figure 1:**
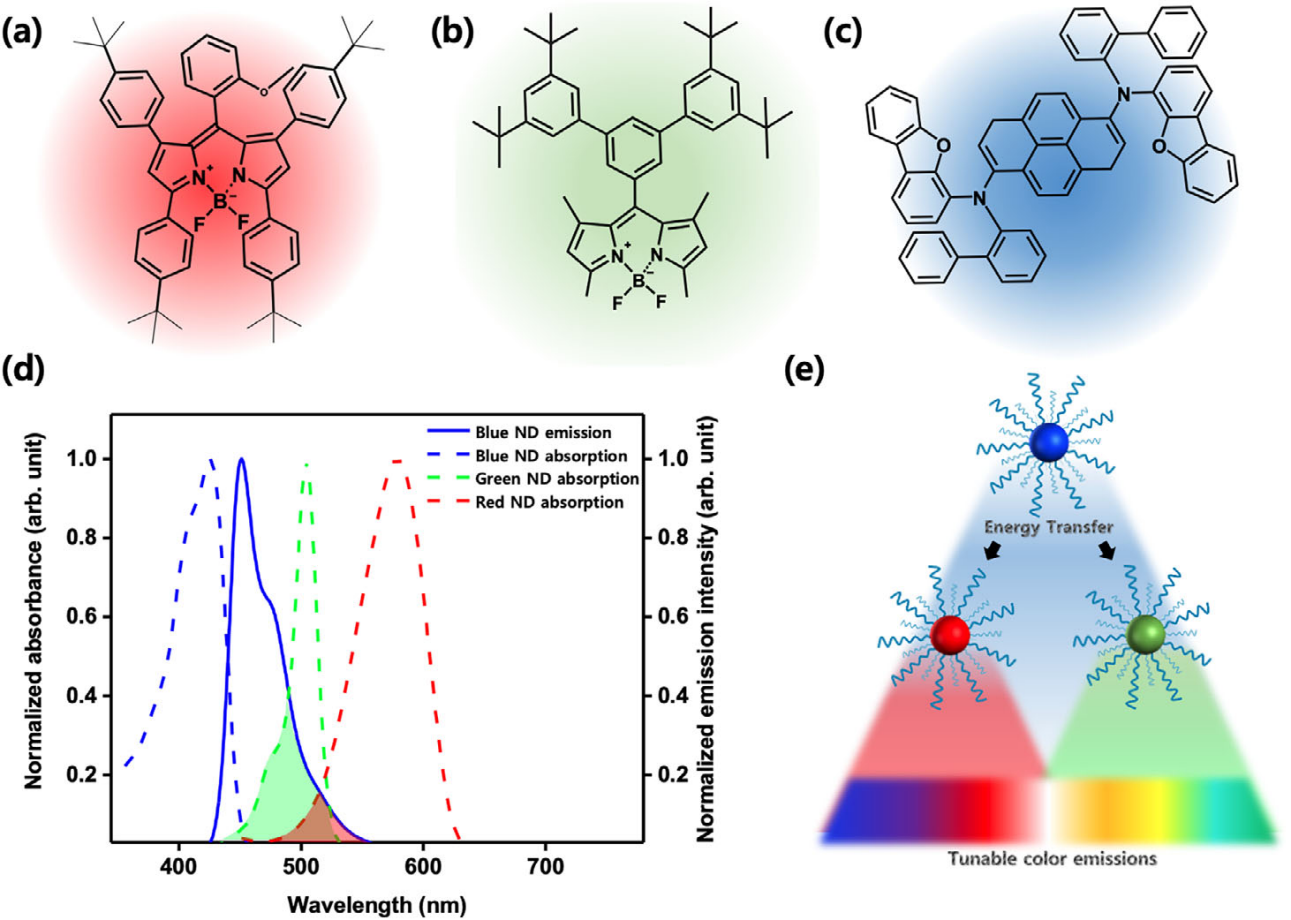
Chemical structure of ND precursors: a) red fluorescent BODIPY, b) green light‐emitting BODIPY, c) pyrene‐based blue light‐emitting material; d) normalized absorption and emission spectra of the aqueous dispersion of organic NDs, each having a concentration of 2.1 mm. e) Conceptual diagram of color‐tunable NDs.

Organic NDs were synthesized using a methodology previously documented in our research. We have prepared three distinct types of nanodots, each exhibiting red, green, and blue emission, utilizing a precipitation method assisted by a surfactant (Triton‐X100). For red NDs (R), we used the same BODIPY‐based dye (**Figure** **1**a) that was used in our previous study,^[^ ^20^ ^]^ a green light‐emitting BODIPY‐based material was used to prepare green NDs (G) (Figure 1b).^[^ ^23^ ^]^ For blue NDs (B), we used a pyrene‐based emitter reported elsewhere (Figure 1c).^[^ ^24^ ^]^ The final stock concentration of each aqueous dispersion was kept at 2.1 mM. The stock dispersion was further diluted to 0.5 mm for low‐concentration experiments (such as DLS and UV–vis turbidity experiments), film processing, and other spectroscopic studies.

### 2.2 Characterization of ND Aqueous Dispersion

The aqueous dispersion of CTONDs was prepared by systematically blending precise amounts of blue‐emitting energy donors with green and red fluorescence‐emitting energy acceptor NDs in varying ratios. The absorption spectra (Figure 1d) of both the green and red ND dispersions exhibited significant overlap with the emission spectra of the blue ND dispersion within the range of 436 to 530 nm for green NDs and 471 to 555 nm for red NDs. This overlap facilitates FRET.^[^ ^22^, ^25^ ^]^ The emission spectra of the blue NDs were generated by exciting them with a light‐emitting diode (LED) at 400 nm wavelength, which doesn't overlap with the absorption spectra of the green and red NDs. Therefore, the energy transfer can be attributed to the emission of the blue NDs. The energy transfer process is illustrated by the conceptual diagram in Figure 1e.

**Figure 2:**
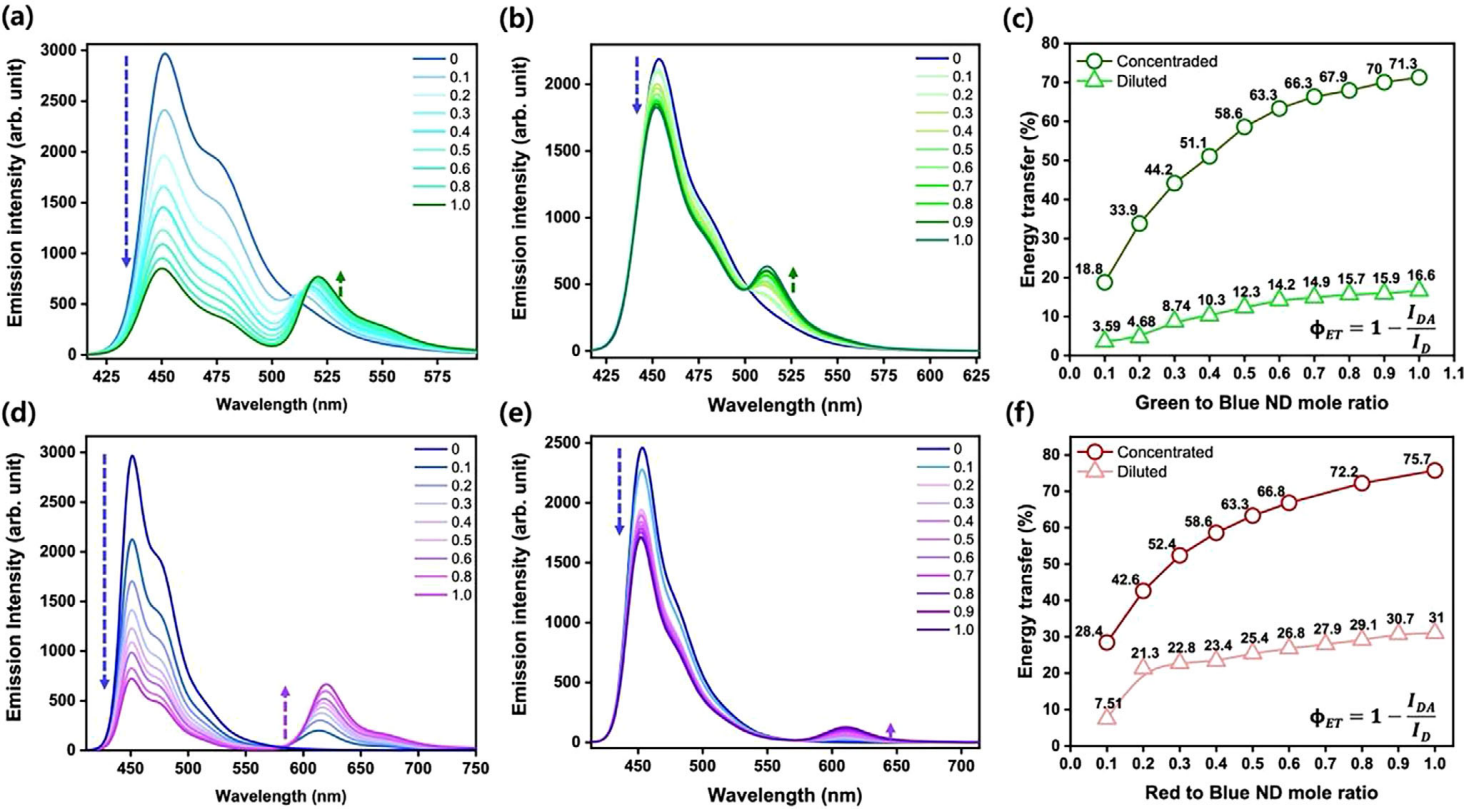
Emission spectra of blue‐green binary ND dispersion a) at high concentration (2.1 mm), b) at lower concentration (0.5 mm); c) energy transfer efficiency curve of blue‐green binary ND mixtures with variable green to blue molar ratio; Emission spectra of blue‐red binary ND dispersion d) at high concentration (2.1 mm), e) at lower concentration of (0.5 mm), f) energy transfer efficiency curve of blue‐red binary ND mixtures with variable green to blue molar ratio.

The fluorescence spectra of the binary ND systems for both red and green NDs exhibited two sets of peaks. The unshifted peak at 451 nm was assigned to the emission of blue NDs, while the shifted peaks at (513–520) nm (**Figure** **2**a) and (613–683) nm (Figure 2d) corresponded to the emission peaks of green and red NDs, respectively, due to FRET. The intensity of the blue ND peak decreased rapidly, whereas the intensity of the green and red ND peaks increased gradually with the incremental addition of green and red NDs separately. Additionally, the absorption of blue ND dispersion at 426 nm remained nearly constant, while the absorption of green and red NDs at 504 and 580 nm, respectively, increased gradually upon the separate addition of energy acceptor NDs (Figure S1a,b, Supporting Information). This suggests the absence of any ground‐state interactions between donor and acceptor NDs. These results imply partial FRET from blue NDs to green and red NDs, respectively. Because of these energy transfer phenomena, we observed a change in the color of the NDs from blue to cyan and blue to purple as the concentration of acceptor NDs increased.

To check the presence of non‐radiative energy transfer and distinguish it from radiative reabsorption, we conducted TRPL measurements on pristine blue light‐emitting NDs and their binary mixtures with green and red NDs. In aqueous dispersion (0.5 mm), the average PL lifetime of the blue donor decreased slightly from 3.77 ns to 3.69 ns (blue‐green) and 3.71 ns (blue‐red), indicating FRET. These shortened lifetimes confirm a non‐radiative mechanism, as reabsorption would not affect the donor decay time. Similarly, green donor NDs showed a decrease from 7.61 ns to 7.13 ns in the presence of red acceptors (Figure S2, Supporting Information). These results confirm a partial non‐radiative energy transfer in aqueous dispersion, where micelle‐based assembly ensures nanoscale proximity of donor and acceptor dyes.

The energy‐transfer efficiencies (ϕ_ET_) were calculated from the emission spectra,^[^ ^5^ ^]^ wherein the fraction of absorbed energy transferred to the acceptor was experimentally measured as a ratio of the fluorescence intensities of the energy donor in the absence (I_D_) and presence of the energy acceptor (I_DA_). In the case of high concentration of the NDs (2.1 mm), an efficiency exceeding 18% was achieved by adding only a 0.1‐mole ratio of green ND to blue ND dispersion. Conversely, the efficiency surpassed 28% when red NDs were added to blue NDs separately at the same molar ratio. Further addition of energy donor NDs resulted in exponential increments in both the red and the green NDs, ultimately reaching ϕ_ET_ values of 71.3% and 75.7%, respectively.

We conducted additional investigations into this phenomenon using lower concentrations of aqueous ND dispersions for both green and red fluorophores (Figure 2b,e). For this experiment, the ND dispersions were prepared at a concentration of 0.5 mm by diluting the stock dispersions. At this reduced concentration, the spatial separation between the energy donor and acceptor NDs would increase compared to the denser ND pairs found in concentrated dispersions. As anticipated, the FRET efficiency of these diluted NDs was notably lessened compared to the original dispersions. The comparison of energy transfer efficiency between concentrated and diluted NDs is illustrated in Figure 2c,f. Notably, the diluted NDs displayed a comparable trend in energy transfer to the concentrated ND pairs, achieving maximum efficiencies of 16.6% and 31% for green and red NDs, respectively. Furthermore, we explored the energy transfer dynamics in these aqueous dispersions by aging both of the NDs for 30 days (Figure S3, Supporting Information). We observed a gradual increase in energy transfer at 10‐day intervals for both the red and green NDs compared to the energy donor blue NDs. This increment in FRET efficiency in the aged dispersions could be attributed to the gradual aggregation of the NDs over time. Specifically, the energy transfer increased from 16.6% to 46.6% for the green NDs and from 31% to 47% for the red NDs.

To visualize and comprehend the color change from human eye perception, we calculated CIE color coordinates (Figure S4, Supporting Information) using the emission spectra of the binary mixed NDs system. Cyan color (0.184, 0.409) emitting NDs (1.0 G: B) was prepared by maintaining a 50:50 molar ratio of blue and green NDs whereas, the magenta (0.315, 0.177) emitting NDs were prepared by adding 30 mol % of red to 70 mol % of blue NDs (denoted as 0.43 R: B on the CIE plot). As the blue NDs could transfer energy to both green and red NDs due to spectral overlap (Figure 1d), we created a ternary mixture encompassing all three colored NDs, resulting in an array of colored emissions. By adjusting the molar ratio of the ternary components, we achieved ND dispersion of nearly perfect white (0.3106,0.3164) fluorescence emission. Moreover, we observed energy transfer owing to the spectral overlap between green NDs' emission and red NDs' absorption (Figure S5, Supporting Information), which expanded the CIE gamut by generating orange (0.486, 0.489) and lime green (0.323, 0.634) colored emissions denoted by 0.05 R: G and 0.25 R: G respectively. Consequently, we successfully achieved a broad spectrum of colors.

### 2.3 Morphology of the CTONDs

**Figure 3:**
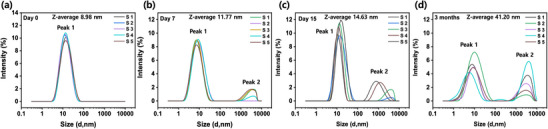
a–d) Intensity‐weighted size distribution profiles of CTOND dispersions measured on Day 0 (freshly prepared), Day 7, Day 15, and 3‐month‐old samples.

To investigate the colloidal stability and aggregation behavior of CTONDs in aqueous dispersion over time, we conducted time‐resolved DLS measurements for a ternary mixture of CTONDs (0.5 mm) across multiple time points (0 day, 7 days, 15 days, and 3 months). DLS results in **Figure** **3** revealed that freshly prepared nanodots exhibited a narrow, monodisperse size distribution with a Z‐average of ≈9 nm and low polydispersity (PI = 0.20), indicating well‐dispersed micelle‐like assemblies. By day 7 (Figure 3b), a secondary population began to appear in the micrometer range (≈3160 nm), and this trend intensified over the period of 15 days (Figure 3c). After three months, the average Z‐average of the dispersion increased to ≈41.2 nm, and the emergence of a prominent secondary peak between 2000 and 3300 nm (Peak 2) accounted for ≈27% of the scattering intensity (Figure 3d). This clear shift to a bimodal size distribution confirms that the nanodots undergo slow, time‐dependent aggregation, likely due to micelle fusion or coalescence. The DLS size measurement data are summarized in Table S1 (Supporting Information).

To further validate these observations, we monitored the CTONDs having the same concentration of 0.5 mm using UV–vis spectroscopy in a non‐absorbing region (700–800 nm) to assess turbidity caused by light scattering. As shown in Figure S6 (Supporting Information), the baseline absorbance in this region gradually increased from Day 0 to Day 20. Since none of the fluorophores absorb in this range, the increase in signal directly correlates with enhanced light scattering due to particle growth and aggregation. The magnified spectra reveal a progressive upward shift in absorbance values, particularly beyond 725 nm, which is characteristic of increasing turbidity caused by larger colloidal assemblies.

**Figure 4:**
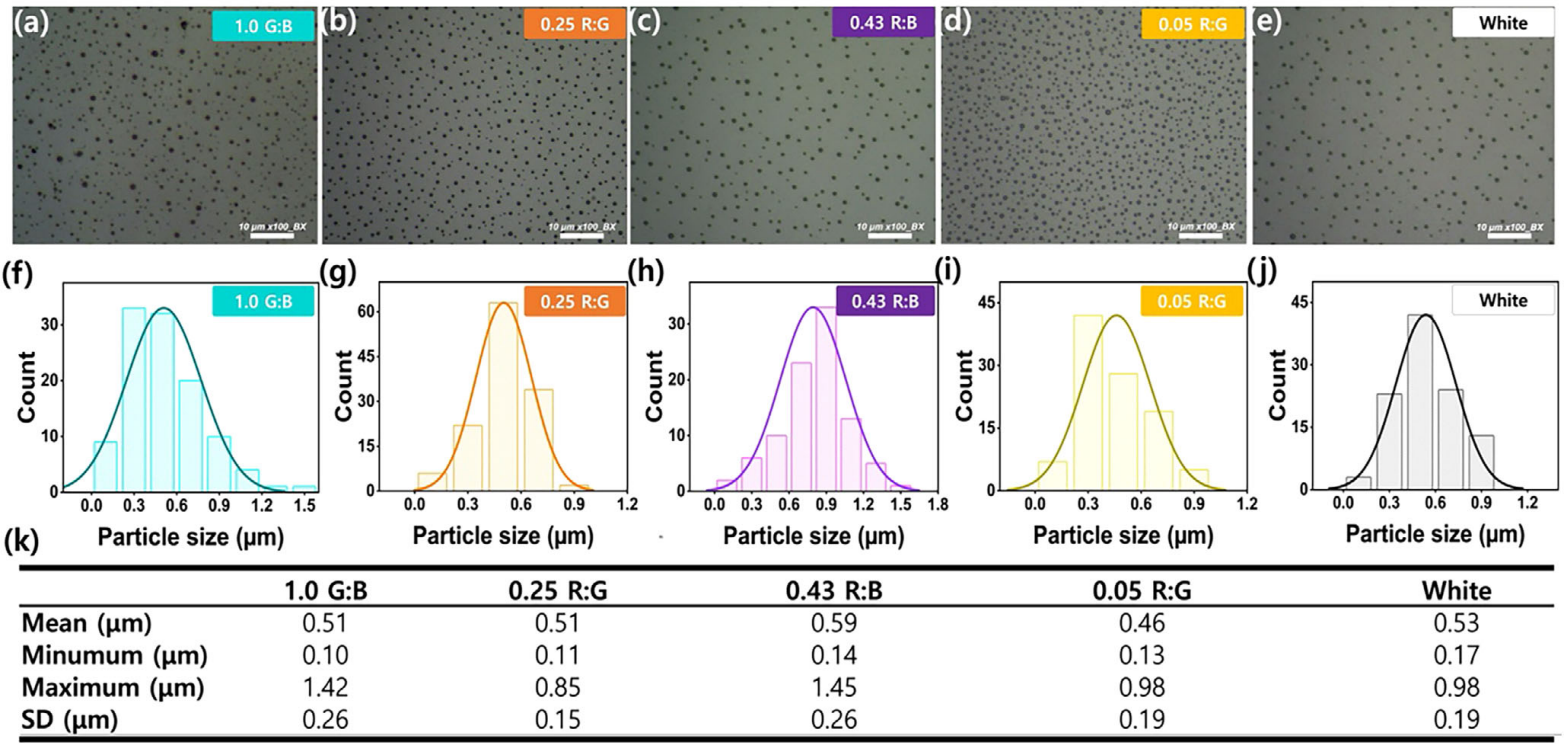
Optical microscopy images of a) 1.0 G: B, b) 0.25 R: G, c) 0.43 R: B, d) 0.05 R: G, e) white NDs film; Histograms of f) 1.0 G: B, g) 0.25 R: G, h) 0.43 R: B, i) 0.05 R: G, j) white NDs film, k) Summary of NDs size distribution.

Next, we checked the size and shape of the CTONDs in dry states using optical microscopy (**Figure** **4**; Table S2, Supporting Information). In our previous study, we extensively investigated the surfactant's influence on the morphology of the single‐colored NDs, finding their average size range from 120 to 200 nm on dry films. However, in the new mixed ND system, where the single‐colored NDs were prepared separately and mixed, the average particle size appeared to range from 450 to 600 nm. This relatively larger size can be attributed to aggregation within the mixed ND system, evident from the DLS analysis. Our earlier work also observed that the NDs in aqueous dispersion tend to aggregate over time. Therefore, it is plausible that in the mixed ND system, individual NDs of the fluorophores fuse with neighboring NDs, leading to bulkier particles typically within an hour in the solid state.

**Figure 5:**
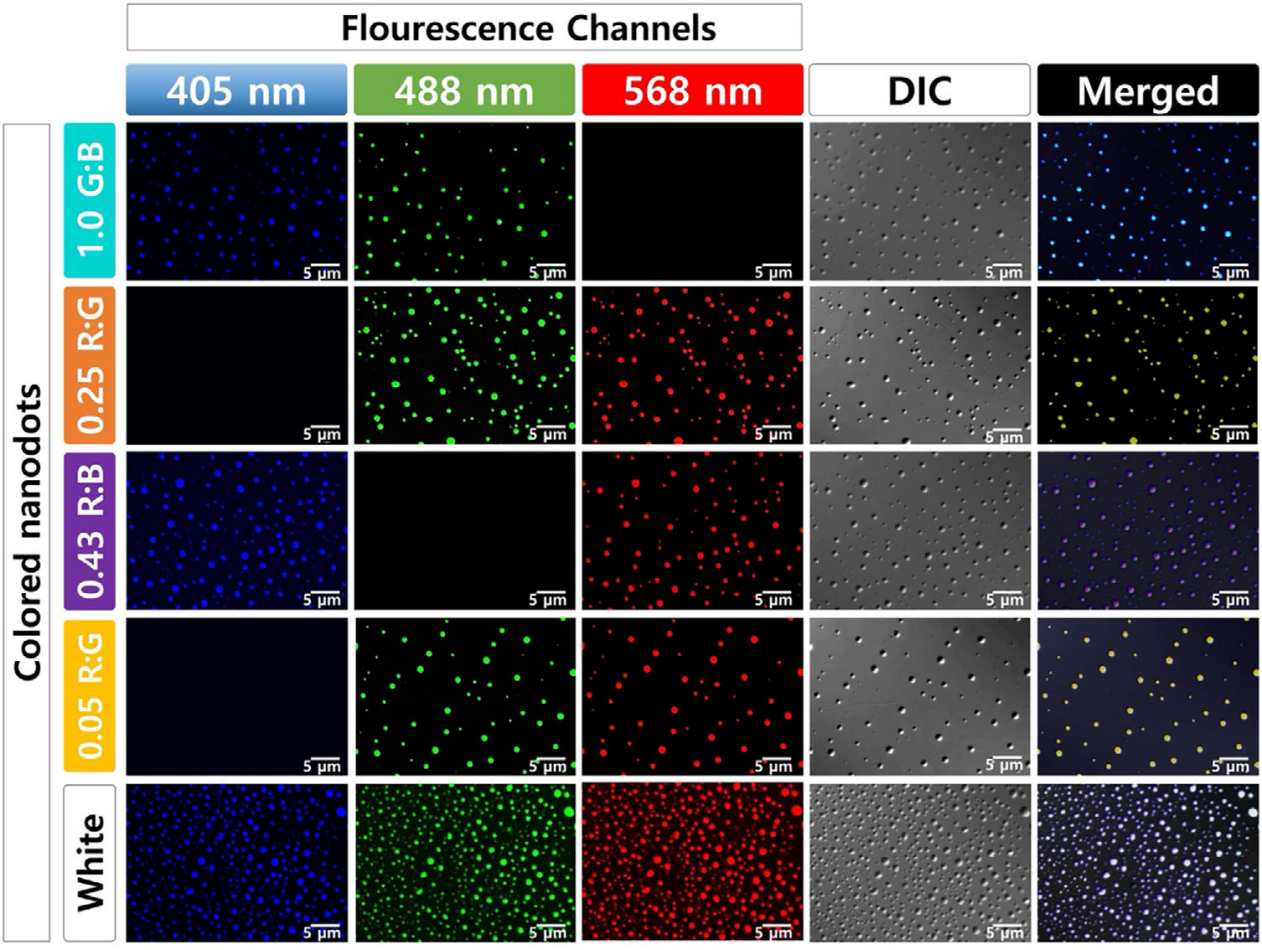
Confocal microscopy images of different binary and ternary CTOND formulations.

To better understand the aggregation processes in the mixed NDs system, we conducted confocal microscopy (**Figure** **5**), where the spatial distribution of each fluorophore in the system can be visualized. The confocal microscope images reveal that due to aggregation and possible diffusion of nanoparticles into one another, larger clusters, ≈500 nm in size on average, have formed. In general, individual NDs initially have sizes around 100 to 150 nm in the solid state, but the aggregation process has significantly increased their size. FRET is strongly distance‐dependent, and aggregation between nanodots with different fluorophores is expected to facilitate energy transfer;^[^ ^26^ ^]^ with blue‐emitting energy donor nanoparticles transferring energy to green and red‐emitting acceptors, and green‐emitting energy donor nanoparticles transferring energy to red‐emitting ones. Energy transfers resulted in shifts in the observed emission spectra, enhancing the overall fluorescence intensity in specific regions and allowing the perceived emission color to be adjusted over a wide range.

The CTONDs are visible in their respective fluorescence channels (blue‐405 nm, green‐488 nm, and red‐568 nm) and the differential interference contrast (DIC) image, indicating that aggregation and diffusion are widespread across the samples. Notably, all the individual particles in mixed samples show emission in multiple wavelengths; in the absence of diffusion, each particle would show only one emission color corresponding to one type of fluorophore. For instance, in the 1.0 G: B binary ND mixture, all of the particles in the image showed cyan emission (corresponding to both green and blue emission from each particle). For ternary mixtures of R, G, and B particles, we observed all three colors (white) emission from each particle.

**Figure 6:**
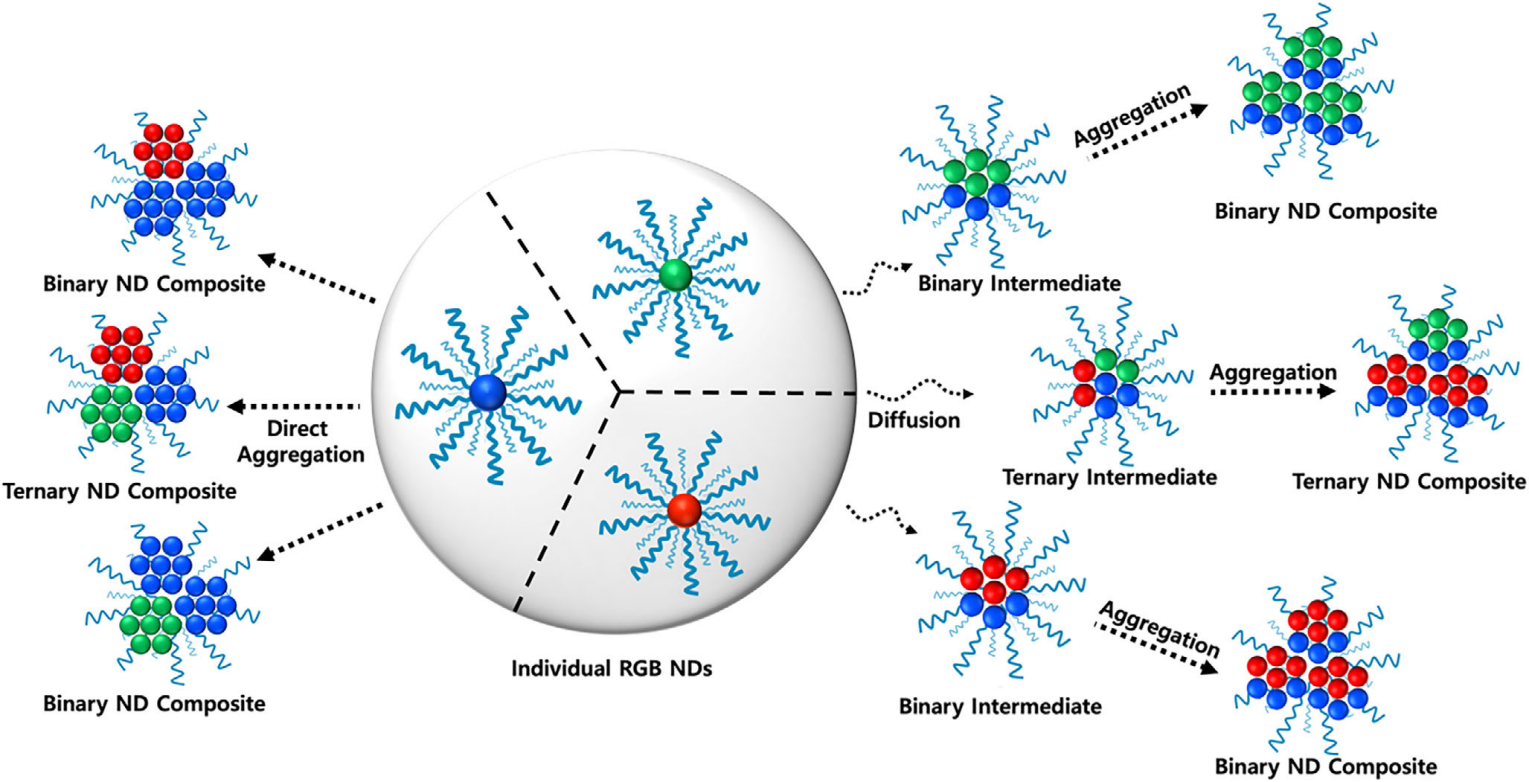
Plausible mechanisms of the formation of the CTOND composites.

The merged images of the composite colors show the interaction between different types of nanoparticles more clearly. Overlapping colors in the merged images reveal that all the particles show relatively uniform emission colors from multiple wavelengths, indicative of the proximity of different fluorophores. This might result from either the diffusion of fluorophores between two nanoparticles into each other, followed by aggregation of these intermediate nanoparticle clusters into larger composite particles, or direct aggregation of different individual NDs into nanoparticle composites, depicted in **Figure** **6**. These interactions lead to new emission properties that are different from those of the individual nanoparticles composed of single fluorophores before mixing, providing insights into the dynamic processes occurring within the samples. For example, all the particles in the 1.0 G: B ND mixture show similar cyan emission as a result of energy transfer, where their individual ND constituents showed green and blue emission in their respective channels. We have also checked the DIC images of the NDs, which provide clear structural details, showing the nanoparticles' physical morphologies in the dry state, which correlate with the fluorescence data. While confocal microscopy does not provide molecular resolution or direct evidence of FRET, it offers qualitative visual support for the spatial proximity of emissive species and fluorescence color mixing.

### 2.4 Film Formation

Acknowledging the successful energy transfer of the NDs from their binary and ternary dispersions, we next investigated their optical properties in the solid state using films of the ND mixtures on glass substrates to obtain a detailed understanding of energy transfer in the solid state. Since FRET efficiency increases for smaller donor: acceptor separation distances, and the NDs remain closely packed in the solid state, ND films are expected to undergo energy transfer more efficiently than in the dispersion state. To confirm this, we selected a handful of mixed ND systems covering almost all the representative colors within the visible spectra. To prepare the mixtures for film casting, we dispersed them in a solution of PVA matrix and TiO_2_. TiO_2_ was chosen for its light‐scattering properties, while PVA served as an inert, solid matrix for embedding the NDs.

**Figure 7:**
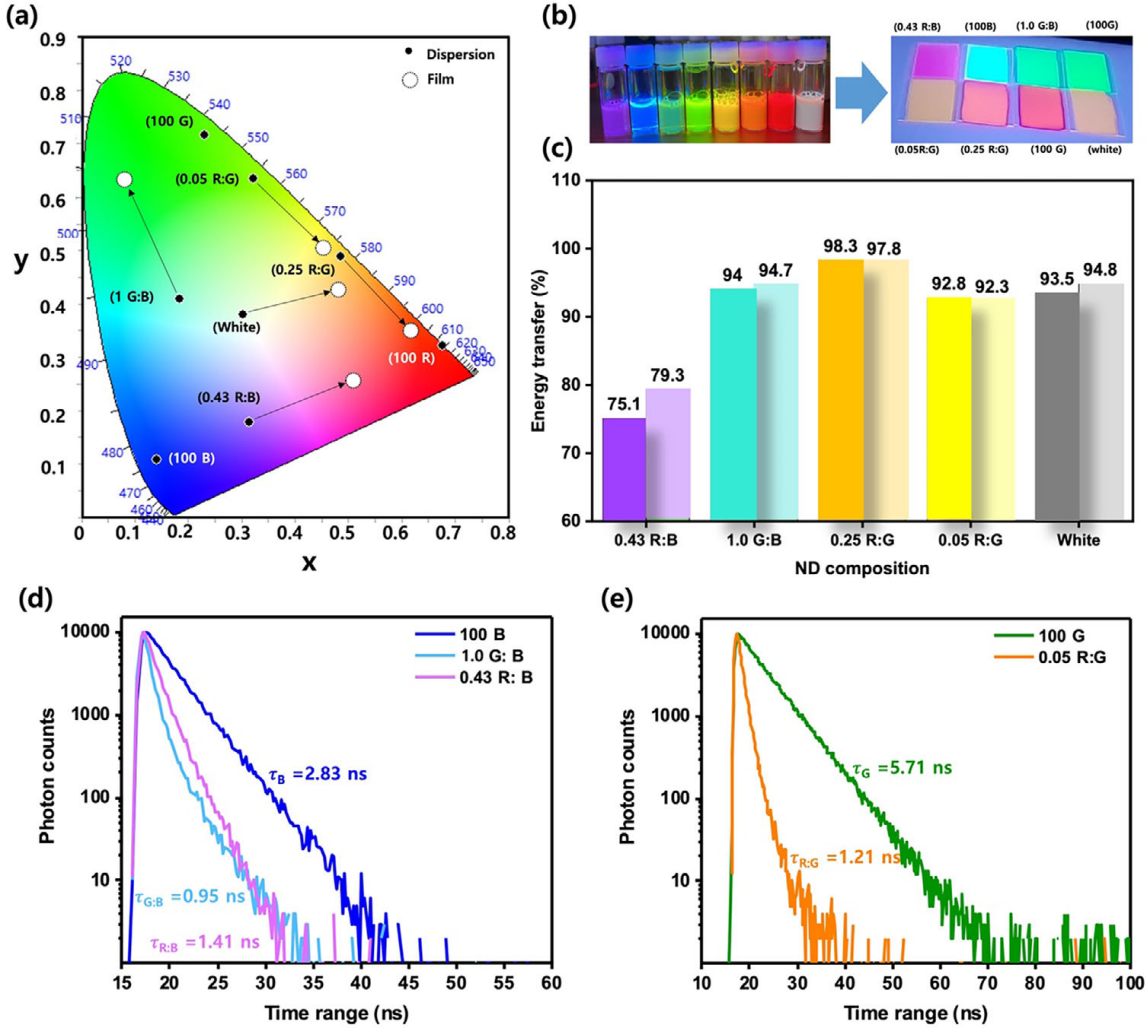
a) CIE color coordinates of ND dispersions and the change in emission color in their film state, the black dot represents the emission color of the NDs in dispersion, and the dotted circle represents their emission colors in film. b) Visual representation of the actual color of the dispersions and the films of the NDs under UV light, from left, 0.43 R: B, 100 B, 1.0 G: B, 100 G, 0.05 R: B, 0.25 R: G, 100 R, and white, c) energy transfer efficiency of the CTOND films; darker colored columns represent the ϕ_ET_ of freshly prepared films and the adjacent lighter colored columns represents. ϕ_ET_ of 4‐month‐old films, d) TRPL decay profiles of 100 B and binary 1.0 B: G and 0.43 R: B CTOND films, e) TRPL decay profiles of 100 G and binary 0.05 R: G CTOND films.

In **Figure** **7**a, the color change in the solid state can be attributed to more efficient energy transfer between the fluorophores in the solid state, as illustrated in the CIE plot. The black dots represent the emission colors of the NDs in dispersions, while white markers signify the emission colors of the solid‐state films. Notably, all mixed NDs displayed substantial shifts in spatial arrangement within the CIE plot. For instance, a binary mixture of green and blue NDs (1.0 G: B) shifted from cyan to the green region, while another mixture of red and blue NDs (0.43 R: B) transitioned from magenta to the purple region. Similarly, binary mixtures of red and green NDs (0.05 R: G and 0.25 R: G) underwent shifts from yellow to orange and from orange to pink, respectively. The color of the resulting white film also shifted toward the orange region on the CIE plot. These observations indicate that more energy transfer occurs in the solid state compared to dispersions, leading to a general redshift of the colors of all the mixed ND films. Actual colors of the final dispersions and films under UV light are shown in Figure 7b, and for direct comparison, their PL and UV–vis spectra for both dispersion and film are provided in the supporting information section (Figures S7 and S8, Supporting Information).

Furthermore, the energy transfer within the films was quantified using established methods described previously,^[^ ^5^ ^]^ which is also shown in Figure S9 (Supporting Information). These figures demonstrate a significant reduction in the blue emission region from the blue NDs, which was absorbed by both green and red‐colored NDs in the 1.0 G: B, 0.43 R: B, and white films, indicating effective energy transfer in the system. Similarly, a reduction in the green emission intensity from the green NDs was observed in the 0.25 R:G and 0.05 R:G films, where the emission was partially absorbed by the red‐colored NDs, further confirming energy transfer along the spectral cascade. Figure 7c provides a comprehensive summary of the energy transfer dynamics observed in the NDs under investigation. The ND films were further analyzed by aging them up to 4 months; in contrast to the mixed dispersions, whose emission colors shifted over time, aged films showed only slight changes compared to freshly prepared films (Figure S10, Supporting Information).

To further evaluate the energy transfer behavior, we have conducted TRPL measurements on these films. The blue energy donor lifetimes in the films were significantly reduced compared to their dispersion counterparts, indicating even more efficient energy transfer. In the blue–green (1.0 G: B) and blue–red (0.43 R: B) system, the blue donor lifetime decreased significantly from 2.83 ns (pristine) to 0.95 and 1.41 ns respectively (Figure 7d), while the green donor in the green–red (0.05 R: G) system showed a lifetime reduction from 5.71 ns to 1.21 ns upon mixing (Figure 7e). These marked decreases suggest that the close packing of nanodots within the solid matrix brings donor and acceptor dyes into tighter proximity, thereby enhancing non‐radiative energy transfer. This result is consistent with the higher FRET efficiencies and stronger emission color shifts observed in the solid films compared to dispersion.

Interestingly, despite the high concentration and close packing of fluorophores in the solid‐state films, we did not observe significant aggregation‐induced quenching (AIQ). This can be attributed to several synergistic design features of our system. First, the fluorophores are pre‐encapsulated within micelle‐like nanostructures formed by Triton X‐100, which spatially isolate dye molecules and suppress π–π stacking in aqueous dispersions. Upon film formation, these nanodots are embedded in a PVA matrix, which acts as a rigid yet inert scaffold that maintains nanodot separation and minimizes interparticle interactions. Furthermore, the energy transfer pathways established between donor and acceptor nanodots (via FRET) help funnel excitons efficiently to emissive sites, reducing the likelihood of non‐radiative decay. Additionally, the bulky side groups on the BODIPY and pyrene‐based fluorophores introduce steric hindrance that might further disrupt aggregation. Together, these factors enable strong and tunable fluorescence in both solution and solid state, without the need for covalent encapsulation or structural modification to combat AIQ.

### 2.5 Application as Color Conversion Layers

Color conversion filters play a crucial role in enabling various lighting and display technologies. They are essential because they transform light from a primary source into different colors, allowing for the display of variable colors or the accurate reproduction of white light. In our research, we investigated the use of CTONDs as filters for color conversion. We achieved this by creating films that incorporated organic nano‐dots dispersed in a carrier polymer, polyvinyl alcohol (PVA), along with TiO_2_ serving as light‐scattering media. To ensure uniformity, we mixed organic ND dispersions with PVA solutions and processed them into consistent films.

**Figure 8:**
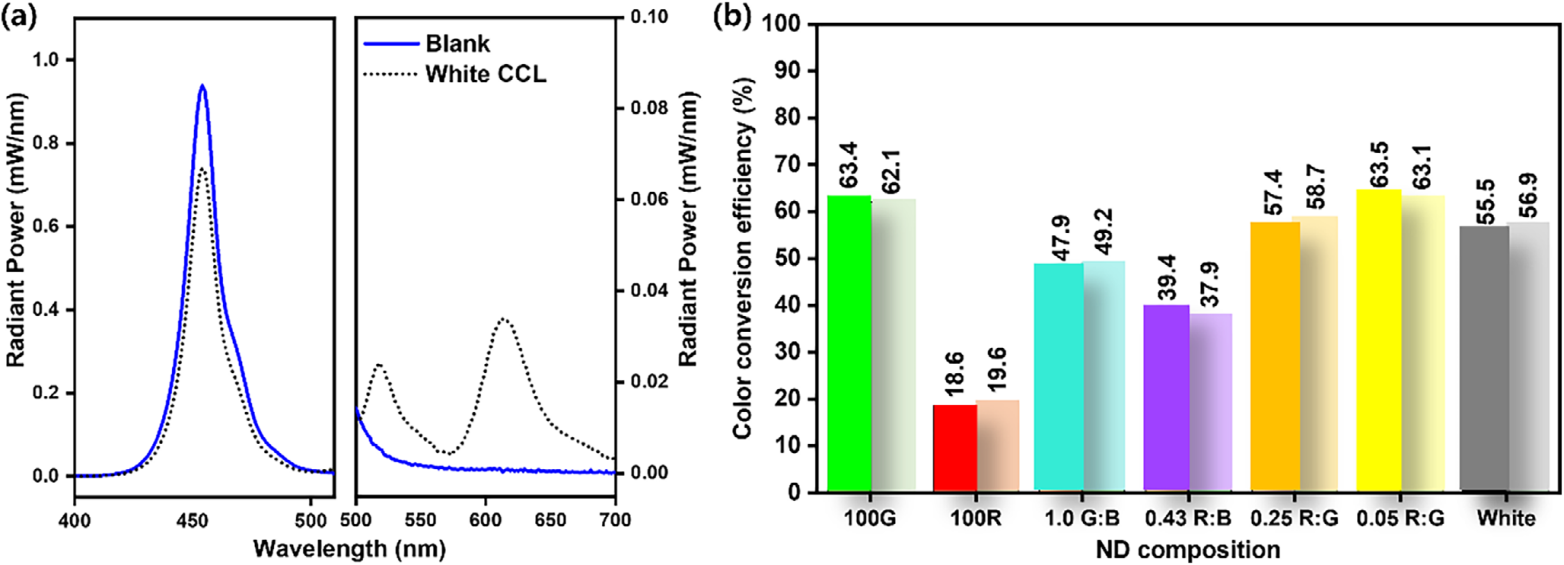
a) CCE of white light emitting ternary ND film used as color conversion layers, b) color conversion performance stability of the different compositions of CTONDs, darker colored columns represent the CCE of freshly prepared films, and the adjacent lighter colored columns represent CCE of aged films.

To excite the films containing organic nano‐dots, we used a blue LED emitting light at a 450 nm wavelength (**Figure** **8**a; Figure S11, Supporting Information). To minimize radiation losses, all experiments were conducted within an integrating sphere. The efficiency of color conversion was assessed by comparing the radiant power of the ND films to that of pristine PVA films of the same thickness without NDs.

We prepared CCLs using different compositions of CTONDs as described in previous experiments.^[^ ^20^ ^]^ Briefly, an LED with a 450 nm emission wavelength was used as a primary light source, simulating the type of light source used in commercial lighting or display applications. Host polymer concentration and film casting procedures were kept identical to the nano‐dot films. Green nanodots labeled as 100 G showed very high color conversion efficiency since their absorption spectra overlap well with the emission spectrum of the LED. On the other hand, red nanodots labeled as 100 R showed inferior performance compared to green nanodots due to the spectral mismatch with the emission wavelength of the LED. CCE obtained from the binary mixtures of nanodots also showed exceptionally high performances due to high energy transfer in their solid state, as mentioned earlier. ND films of 1.0 G: B (a 50–50 mixture of green and blue NDs) resulted in a cyan color with 47.9% CCE. The purple colored NDs, consisting of 30% red and 70% blue, showed the lowest CCE of 39.4% probably due to the inferior ϕ_ET_ of 75.04% compared to all the mixed ND films. In the case of the red and green mixed NDs, films containing the lowest amount of red in their composition (5% red in 95% green) showed the best CCE of 63.5% and resulted in a yellow–orange color. On the other hand, the CCE dropped slightly to 57.4% when the film having 20% red ND in 80% green (0.25 R: G film) was evaluated. The energy transfer of these films also shows that this film exhibited the highest ϕ_ET_ value (98.26%) compared to the film having higher red NDs (0.25 R: G). Finally, white light‐emitting nanodot films showed 55.5% CCE. On the other hand, the reference InP QD CCL used in our previous study converted only 21% of the incident blue light,^[^ ^20^, ^27^ ^]^ To provide a broader context, we compared the photophysical performance of CTONDs with other representative luminescent nanomaterials, including organic nanodots, carbon dots, InP quantum dots, perovskite QDs, and resin‐stabilized QDs (see Table S2, Supporting Information).

We evaluated the stability of these multicolored color conversion films after four months of preservation in the open air. Figure 8b shows the stability data of the nanodot films. Columns containing darker colors represent the initial conversion efficiency of the films, whereas the lighter colored columns represent the CCE of the same films aged for four months. The stability of these nanodots was exceptionally good, showing almost no changes over the aging period. Interestingly, some of the aged ND films showed a slight increase in FRET efficiency compared to freshly cast films. While molecular rearrangement is unlikely in the solid matrix, this improvement may result from gradual polymer relaxation, residual solvent evaporation, or enhanced refractive homogeneity over time. These factors can subtly optimize the local photonic environment, thereby supporting more efficient energy transfer.

### 2.6 Fluorescent Multicolored Ink

Fluorescent inks have found extensive utility across diverse domains, including security printing, smart packaging, information storage, bioimaging, and nano‐electronics. Notably, water‐based fluorescent inks are in high demand due to their environmentally friendly nature. Unlike organic solvent‐based counterparts, these inks are not flammable and do not emit volatile organic compounds, thus facilitating safe storage under standard conditions. The eco‐friendly nature of water‐based fluorescent inks makes them particularly suitable for flexography printing. They possess the advantageous ability to be efficiently dried on highly absorbent substrates with exceptional permeability and can withstand elevated temperatures (Figure S12, Supporting Information). Leveraging the color tunability, stability, and water dispersibility of our NDs, we have identified them as promising candidates for fluorescent inks and security inks.

**Figure 9:**
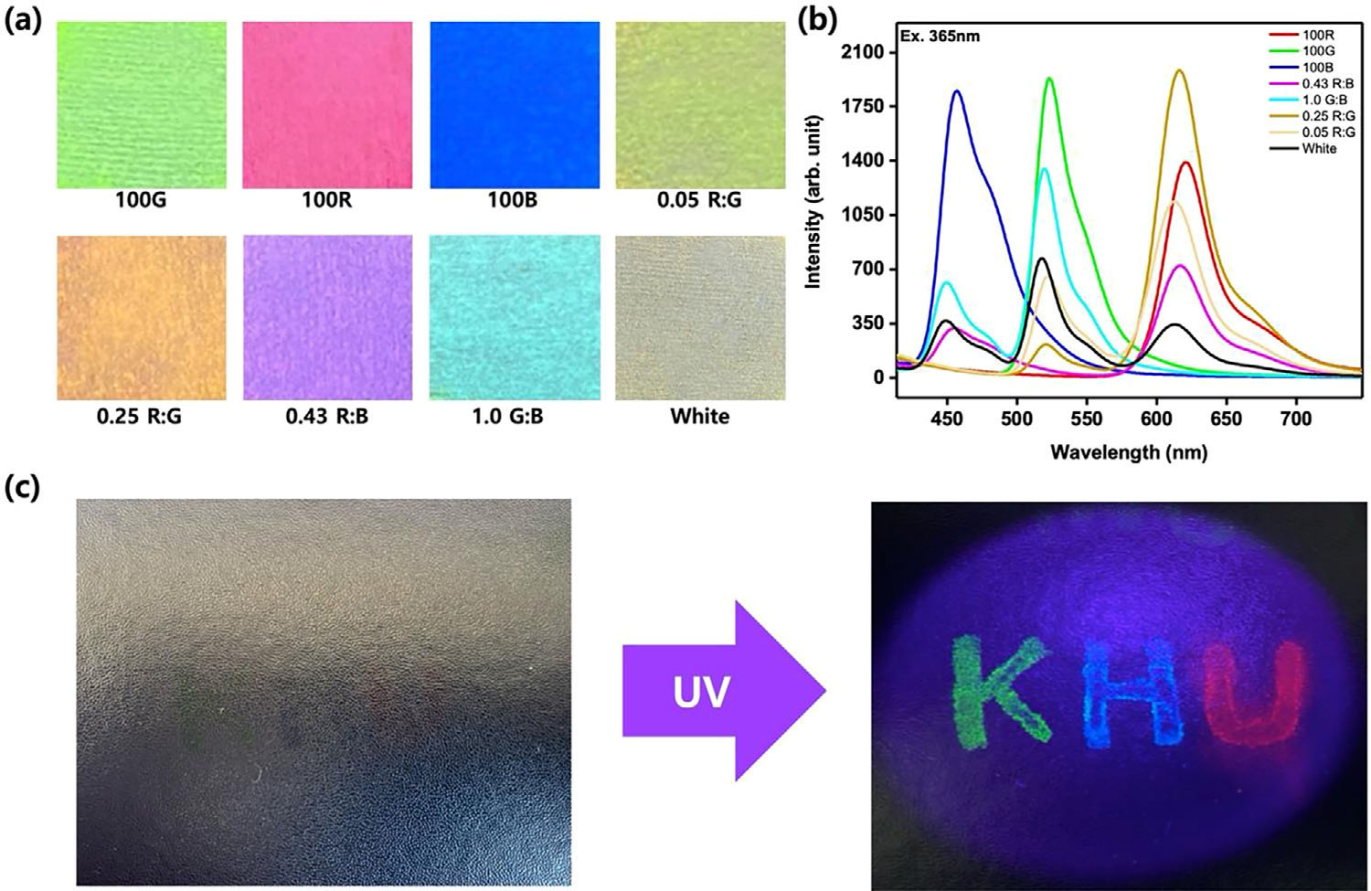
a) White paper stained by different CTONDs under UV light, b) PL spectra of the stained papers, c) Writing on black polyester paper using NDs under normal light and UV light.

To validate CTOND's suitability as fluorescent inks, we conducted experiments with them on ordinary white non‐woven wiper papers, staining them with ND dispersions. The paper used in these experiments was deliberately selected to be of the type without any whitening / brightening agents, which might interfere with the fluorescence emission of the NDs. Subsequently, the stained papers were dried on a hotplate at 60–70 °C to eliminate residual solvents, as illustrated in **Figure** **9**a. We have kept the variation of the NDs similar to the film studies discussed previously. Figure 9b showcases the photoluminescence (PL) emission spectra of the stained papers by the NDs. Notably, the resulting colors of the stained NDs closely matched those observed in the film state. Furthermore, we investigated the potential of these NDs as security inks. To this end, a piece of black polyester‐based paper was stained by writing with one of the ND dispersions (Figure 9c). Under normal light, the writing remained mostly invisible; however, upon exposure to ultraviolet light, the emission color became fully visible, demonstrating its efficacy as a security feature.

## 3 Conclusion

In this work, we developed water‐based color‐tunable organic nanodots that exhibit efficient FRET and stable multicolor emission in both dispersion and solid‐state films. By adjusting the ratios of blue, green, and red emissive fluorophores, we achieved a wide emission gamut, including white light, with energy transfer efficiencies reaching over 90% in films due to close nanodot packing. Time‐resolved PL studies confirmed FRET as the dominant energy transfer mechanism, supported by size‐dependent aggregation trends observed through DLS and UV–vis turbidity measurements. The micelle‐based nanodots retained high photoluminescence without significant aggregation‐induced quenching, owing to the surfactant stabilization, polymer matrix embedding, and molecular design. These CTONDs demonstrated excellent color conversion efficiencies (over 60%), surpassing benchmark InP‐based quantum dots, and exhibited long‐term environmental stability. We further demonstrated their practical viability as fluorescent and security inks on paper substrates, showing robust color retention and high‐temperature resilience. Overall, this study presents a scalable, aqueous‐processable strategy for fabricating tunable emissive nanodots with strong potential for advanced optoelectronic, display, and anticounterfeiting applications.

## 4 Experimental Section

### Materials

All reagents and solvents were purchased from commercial suppliers and used without further purification. Polyethylene glycol tert‐octylphenyl ether (Triton X‐100) and Titanium dioxide (TiO_2_) were purchased from Sigma‐Aldrich Chemical Company and used without any purification. Polyvinyl alcohol (PVA), having an 83–89% degree of hydrolysis, was used for film formation. Cellulose acetate dialysis tubing with a molecular weight cut‐off of 14000 Dalton was purchased from Sigma‐Aldrich. Tetrahydrofuran (THF) was purchased from Tokyo Chemical Industries. 4,4‐difluoro‐2,3,5,6‐tetrakis(4‐(1,1‐dimethylethyl)phenyl)‐4‐bora‐3a,4a‐diaza‐s‐indacene (Red BODIPY), 4,4‐difluoro‐2,3,5,6‐tetrakis(4‐(1‐methylethyl)phenyl)‐4‐bora‐3a,4a‐diaza‐s‐indacene (Green BODIPY), and 1‐(4,4‐difluoro‐4‐bora‐3a,4a‐diaza‐s‐indacen‐8‐yl)pyrene (Blue Pyrene) were synthesized using published procedures.

### Instrumentation

UV–vis absorption spectra were collected using a Shimadzu UV‐3600i Plus UV–vis–NIR Spectrometer. Photoluminescence (PL) spectra were collected using a JASCO FP‐8500 spectrofluorimeter. Absorbance spectra were obtained using a JASCO V‐750 UV–vis spectrometer, and photoluminescence (PL) spectra. The time‐resolved photoluminescence (TRPL) spectra were recorded on a Hamamatsu instrument (C11367). Optical microscope images were taken with an Olympus BX51 microscope. The hydrodynamic diameter of the NDs was measured by dynamic light scattering (DLS) (Marlvern, UK) using NDs having 0.5 m concentration and measured at room temperature. Confocal laser scanning microscopy was performed in an Olympus FLUOVIEW FV3000 equipped with 405, 488, and 561 nm laser channels. Color conversion efficiency was measured using a 6‐inch integrating half‐sphere with a high‐speed LED spectrometer (LE‐5400 Otsuka Electronics) and a Keithley 2401 source meter.

### ND Aqueous Dispersion Preparation

The procedure involved dissolving each of the fluorophores separately into THF solvent to create a 0.5 mm stock solution. Triton X‐100 surfactant was dissolved separately in the same solvent to prepare a 0.1 m solution. Subsequently, 0.1 mL of the fluorophore solution was placed in a vial, and 0.3 mL of the surfactant solution was added. Excess THF was added to maintain a constant total volume of 0.6 mL. Deionized water was then rapidly injected (4.4 mL) as the anti‐solvent into the fluorophore‐surfactant solution to form nano‐dot dispersions, with the total volume maintained at 5 mL. These dispersions were filtered through PTFE syringe filters having a pore size of 200 nm in diameter and subjected to dialysis using cellulose acetate tubes for 12 h to remove any excess surfactant. Finally, the dispersions were concentrated under reduced pressure (≈0.01 Torr) using a Schlenk line to evaporate 90% of the solvent. After vacuum evaporation of the excess solvent, the dispersion was diluted with deionized water to maintain a concentration of 0.5 and 2.1 mm for subsequent experimental analyses.

### Film Preparation

A total of 0.27 mL of mixed NDs dispersion (1 wt%) and 0.019 mL of TiO_2_ water dispersion (15 wt%) were taken in a 5 mL vial and stirred for 20 min at room temperature to get a uniform mixture. Then, 1.2 mL of PVA polymer solution in water (22 wt%) was added to the above‐prepared mixture. After 20 min of stirring, a homogenous polymer matrix with NDs‐TiO_2_ was attained. The films were fabricated on a clean glass substrate with a bar‐coater and dried at 80 °C for 2 h. Finally, it results in a transparent and highly uniform thin fluorescent ND film with a thickness of 20 µm measured with alpha‐step profiler.

## Author Contributions

Y.K. and R.A. contributed equally to this work. Y.K. carried out the color‐tunable nano‐dot synthesis, characterizations, and applications. R.A fabricated and characterized the nano‐dot films and the color conversion filters. Confocal microscopy and DLS measurements were performed by K.A.R. All the experiments and characterizations were conducted under the supervision of B.W. and J.H.K.

## Conflict of Interest

The authors declare no conflict of interest.

## Supporting information



Supporting Information

## Data Availability

The data that support the findings of this study are available from the corresponding author upon reasonable request.
