# Supplementary material for: Color‐Tunable Organic Nano‐Dots: Synthesis and Applications in Color Conversion and Security Inks
Source: Small. 2025 Jul 10;21(46):2505043. doi: 10.1002/smll.202505043 (PMC12632417; doi:10.1002/smll.202505043)
Supplement: Supplementary file 1 — Supporting Information [file SMLL-21-2505043-s001.docx]

Supporting Information

Color-Tunable Organic Nano-Dots: Synthesis and Applications in Color Conversion and Security Inks

Yeasin Khan, Rasheeda Ansari, Khandoker Asiqur Rahaman, Bright Walker**^*^** and Jang Hyuk Kwon**^*^**


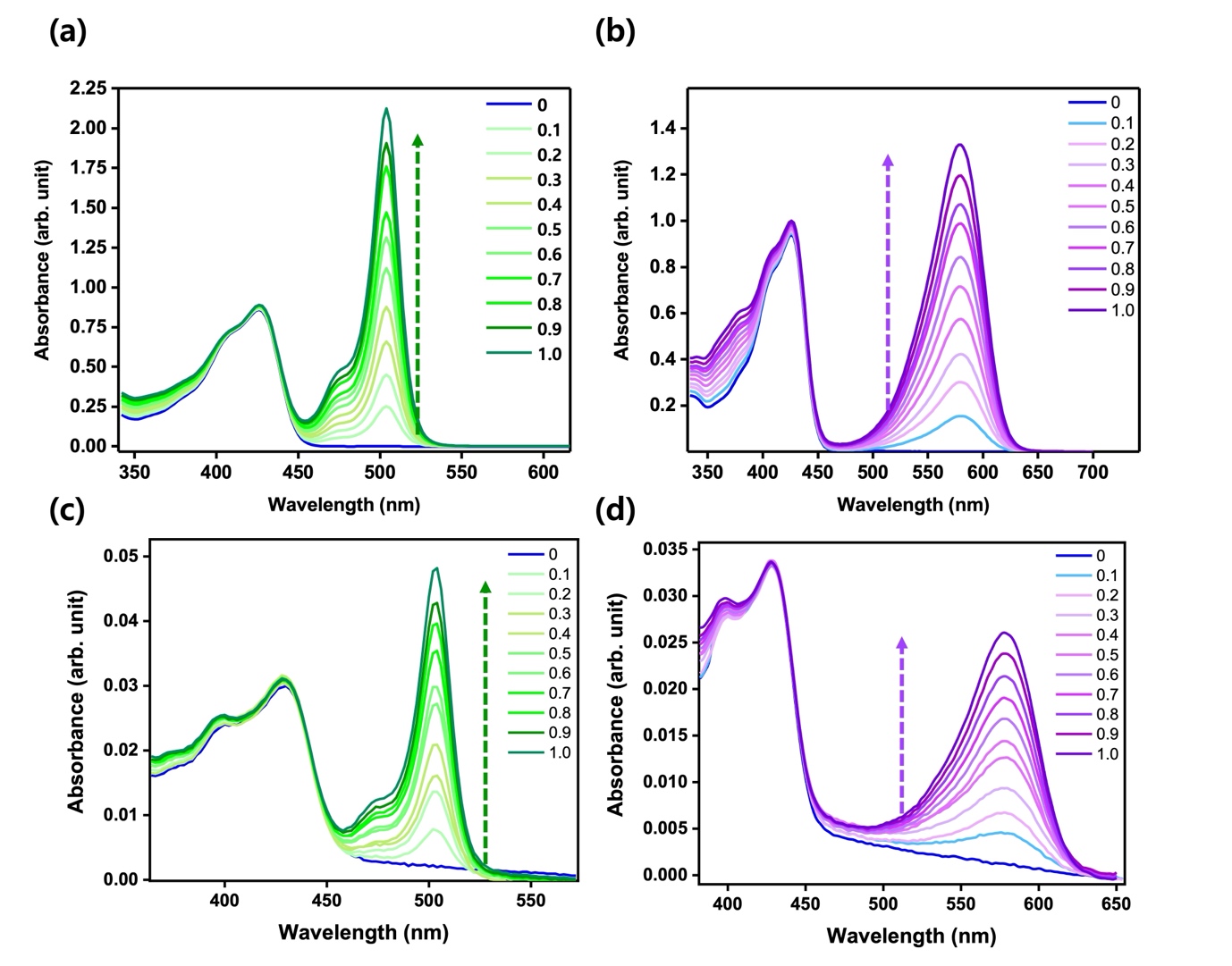


**Figure S1.** (a) Absorption spectra of blue-green binary ND dispersion at concentration of 2.1 mM, (b) Absorption spectra of blue-red binary ND dispersion at concentration of 2.1 mM,; (c) Absorption spectra of blue-green binary ND dispersion at concentration of 0.5 mM, (d) Absorption spectra of blue-red binary ND dispersion at concentration of 0.5 mM.

**
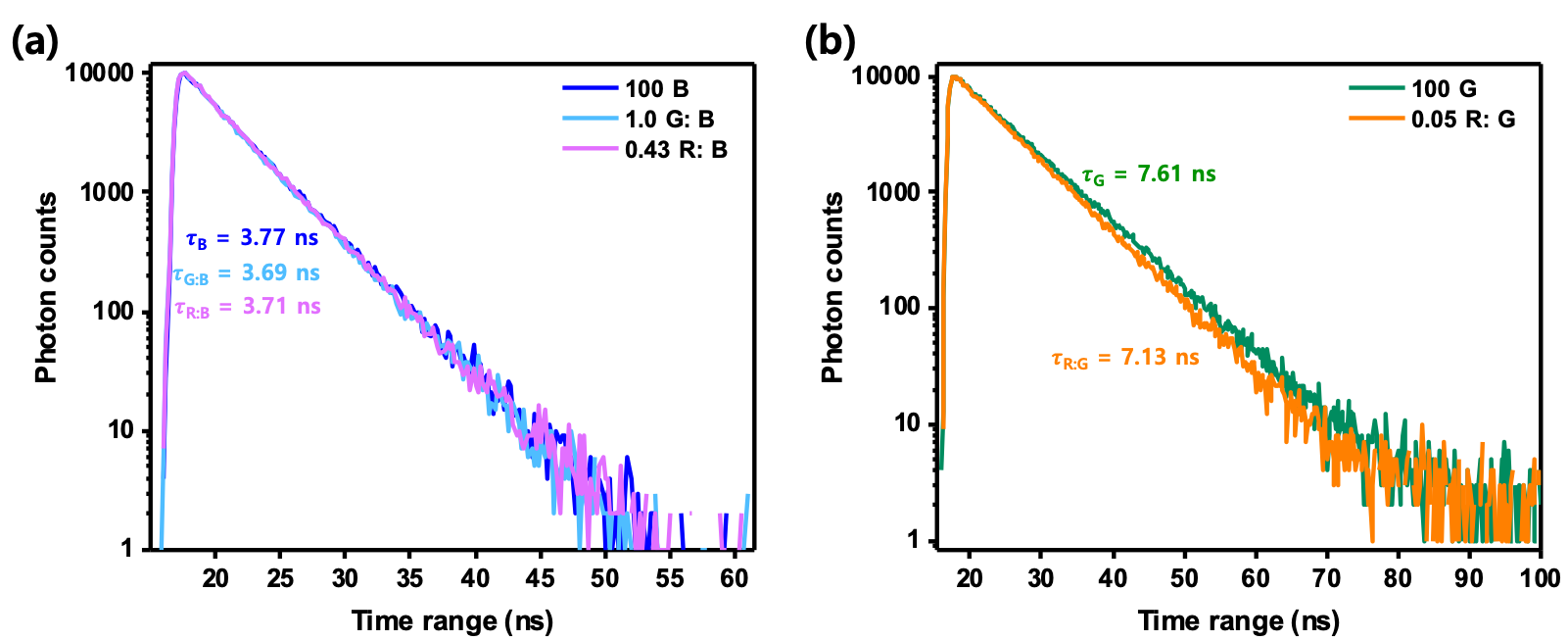
**

**Figure S2.** (a) TRPL decay profiles of 100 B and binary 1.0 B: G and 0.43 R: B CTOND dispersions, (b) TRPL decay profiles of 100 G and binary 0.05 R: G CTOND dispersions.


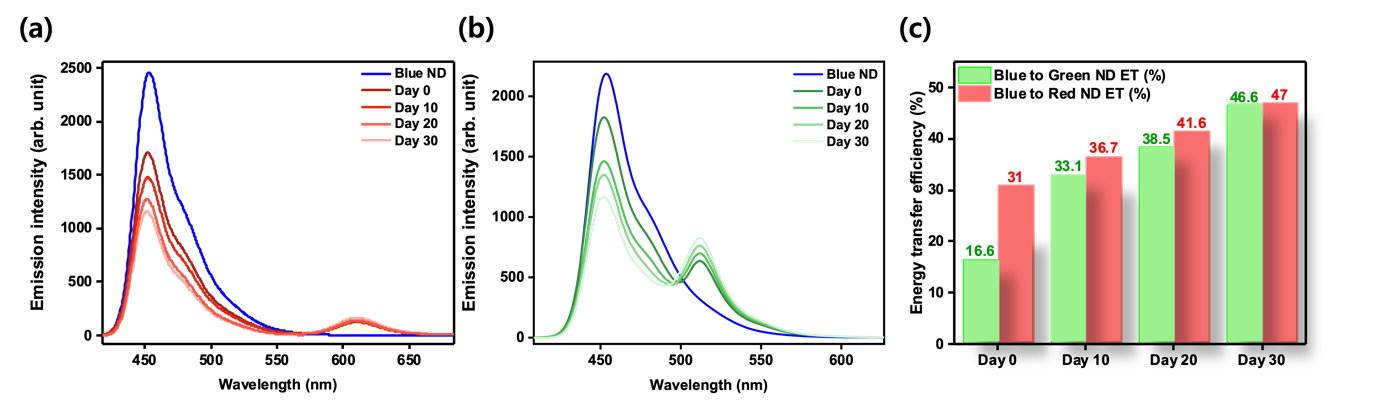


**Figure S3.** Evolution of FRET in aged ND aqueous dispersions. (a) Energy transfer from blue to red ND dispersions, (b) energy transfer from blue to green ND dispersions, (c) comparison of energy transfer increment over time for green and red NDs with respect to energy donor blue NDs.


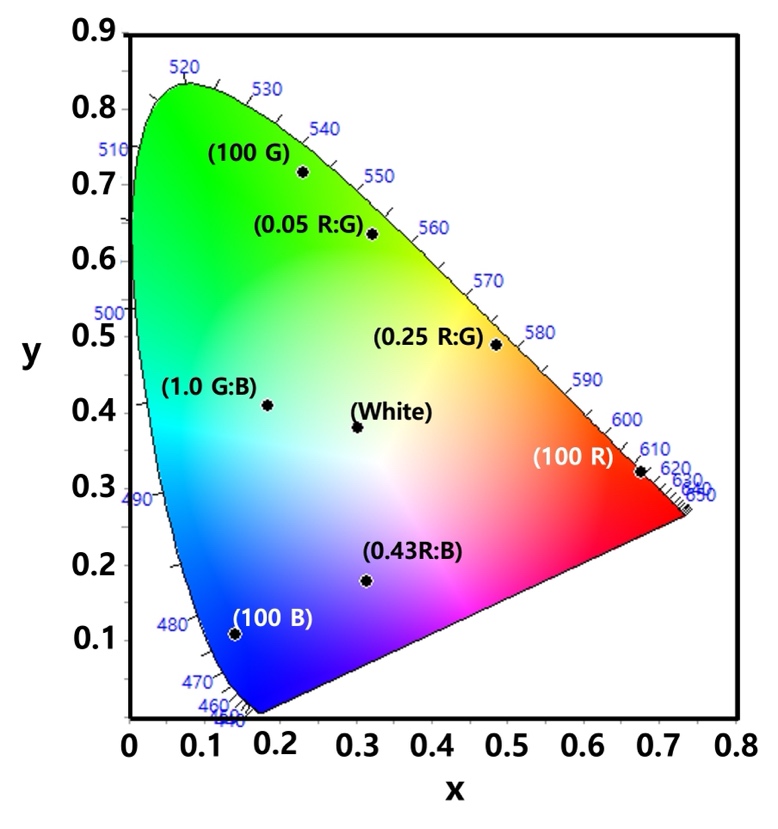


**Figure S4.** CIE color coordinates of various CTOND aqueous dispersions.


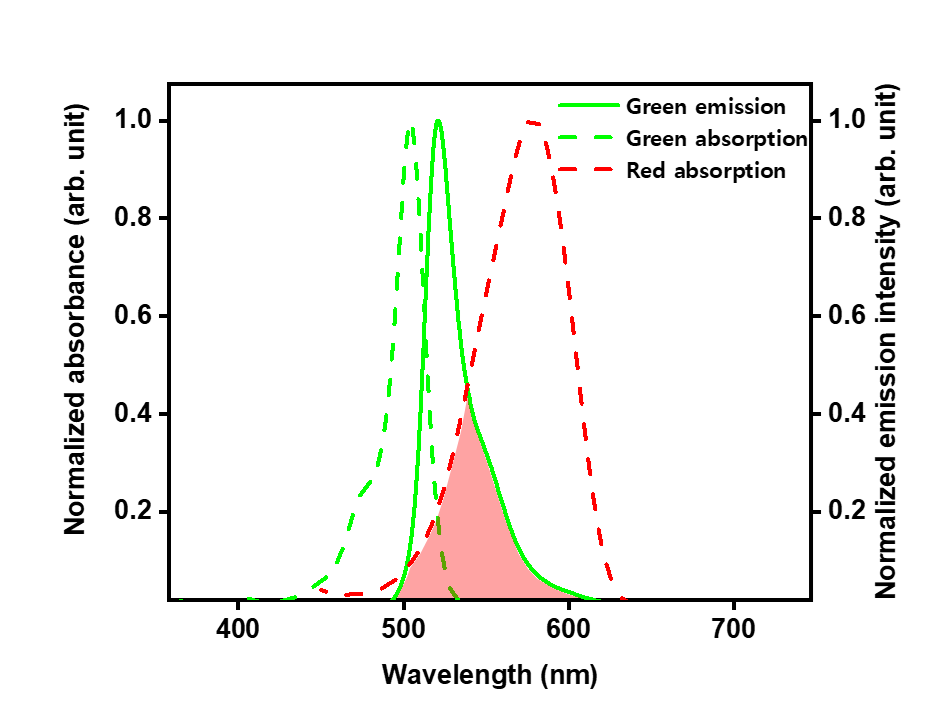


**Figure S5.** Absorption and emission spectra of the aqueous dispersion of green and red emitting organic NDs.


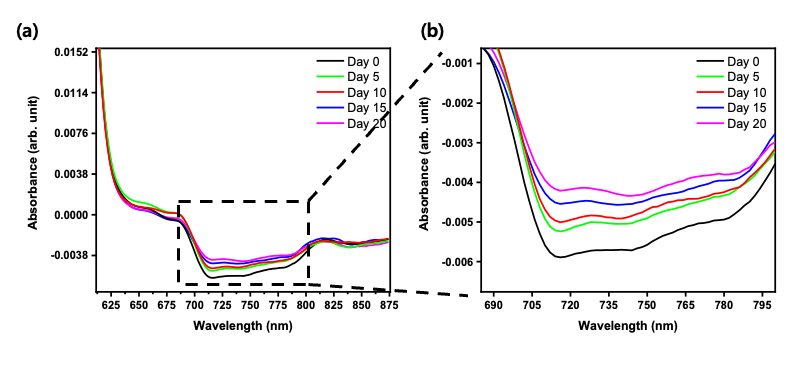


**Figure S6.** Time-dependent UV-Vis turbidity analysis of CTOND dispersion. (a) Absorbance spectra of CTONDs measured over 20 days show increasing baseline absorbance in the 700–800 nm range, where no fluorophore absorption occurs, (b) Magnified view of the baseline region highlights increased scattering from Days 0 to 20, confirming aggregation-induced turbidity in aged dispersions.


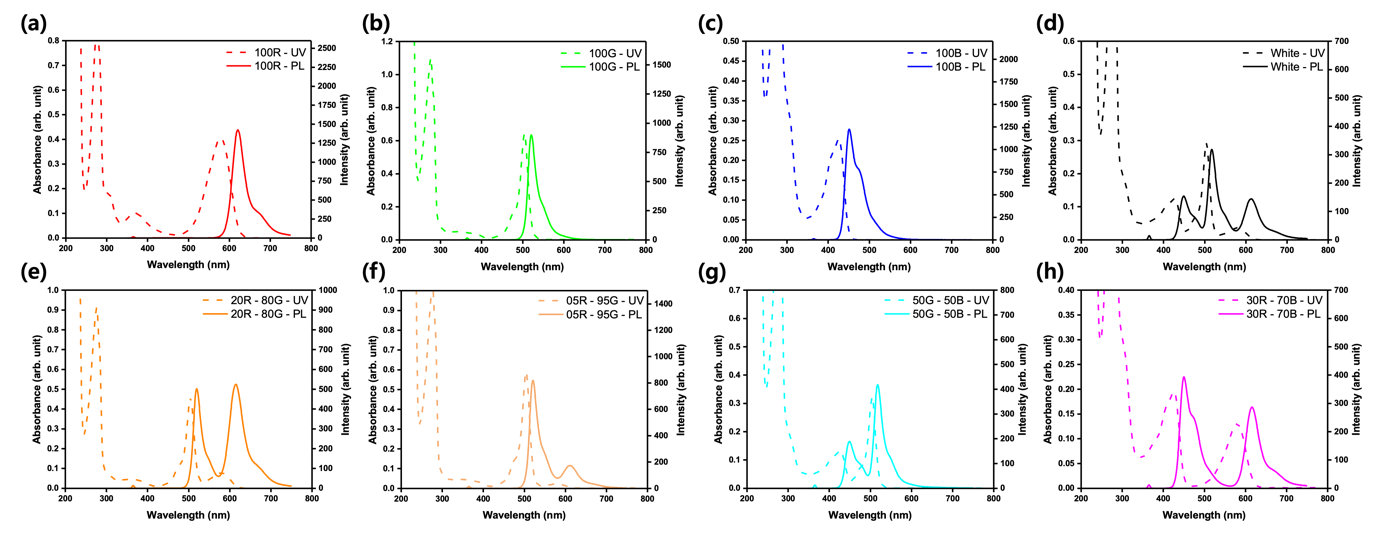


**Figure S7.** PL and UV comparison of CTONDs in water dispersion.


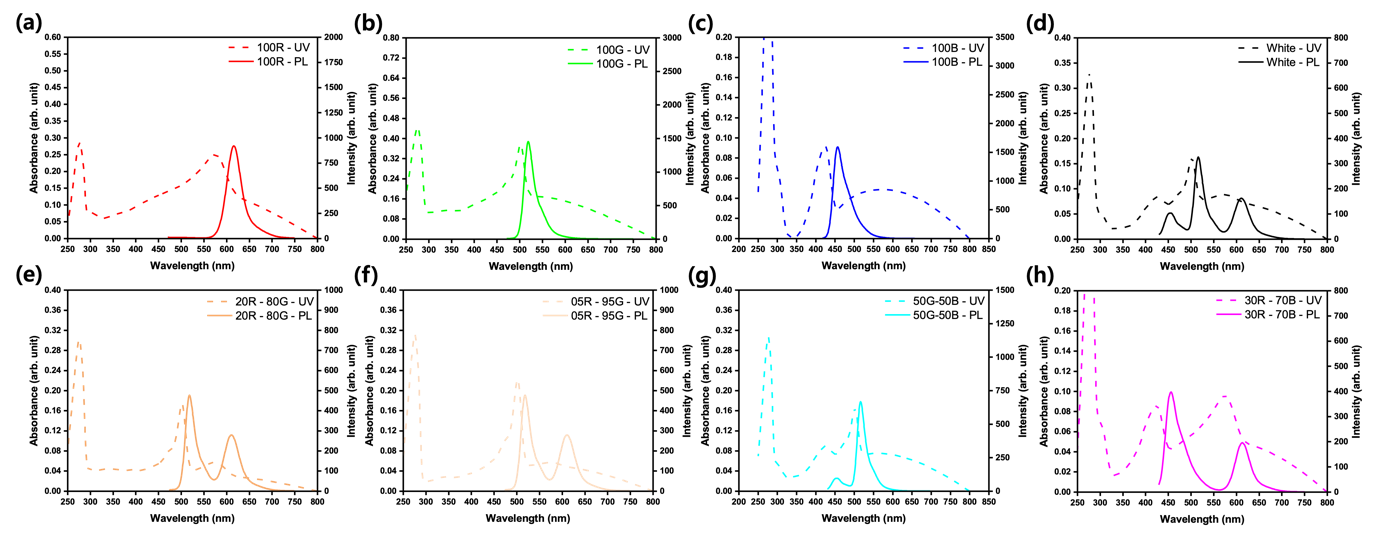


**Figure S8.** PL and UV comparison of CTOND Films.


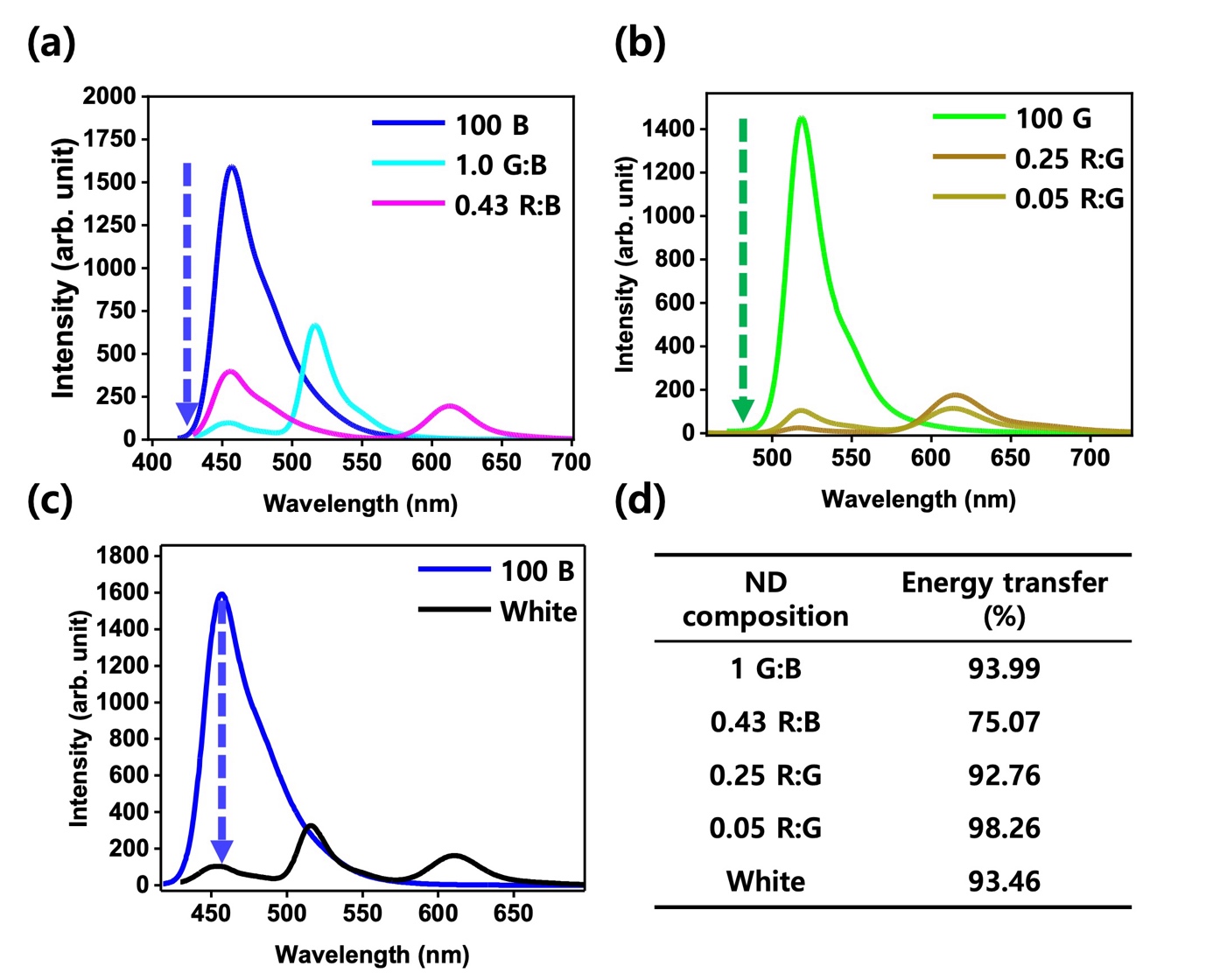


**Figure S9.** (a) PL spectra of 1.0 G:B and 0.43 R:B films compared to pure blue emission, (b) PL spectra of 0.25 R:G and 0.05 R:G compared to pure green emission, (c) PL spectra of white emission compared to pure blue emission, (d) energy transfer in the solid state of different composition of CTONDs.


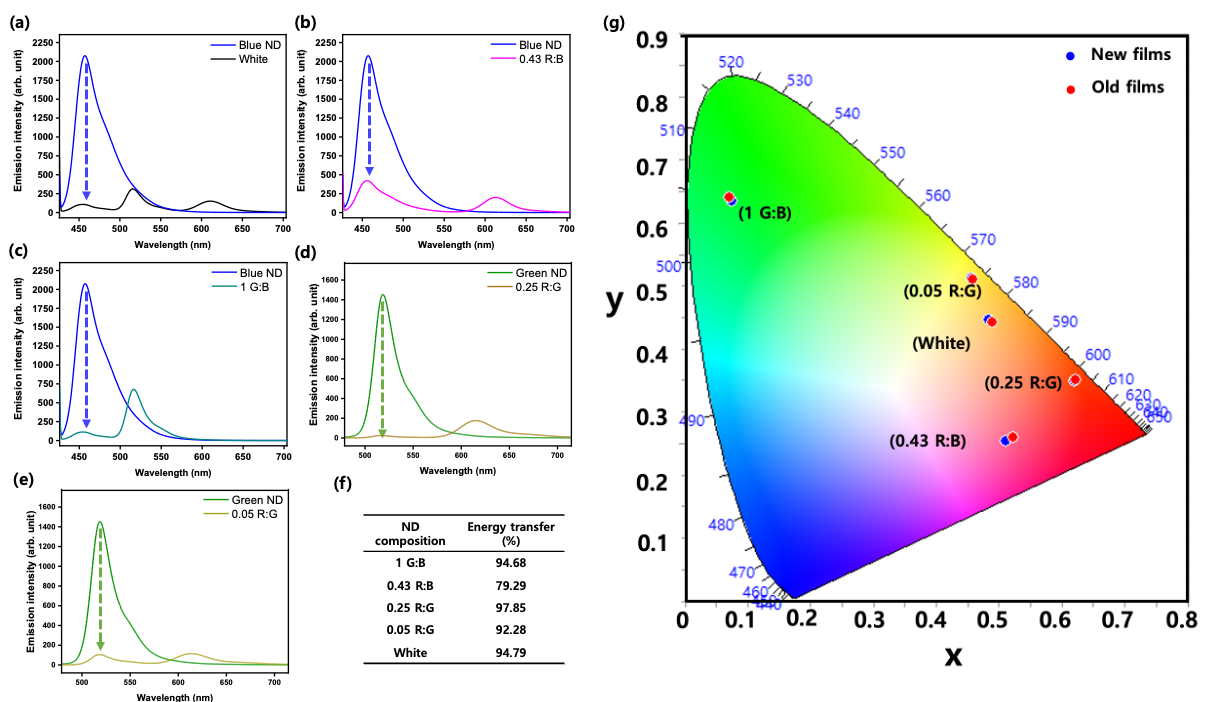


**Figure S10.** PL spectra of over 4 month old film of (a) white, (b) 0.43 R:B, (c) 1.0 G:B compared to pure blue light emitting ND; and (d) 0.25 R:G, (e) 0.05 R:G compared to green light emitting ND; (f) summary of energy transfer of these 4 month old CTONDs. (g) CIE coordinates of freshly prepared and 4 months old CTOND films


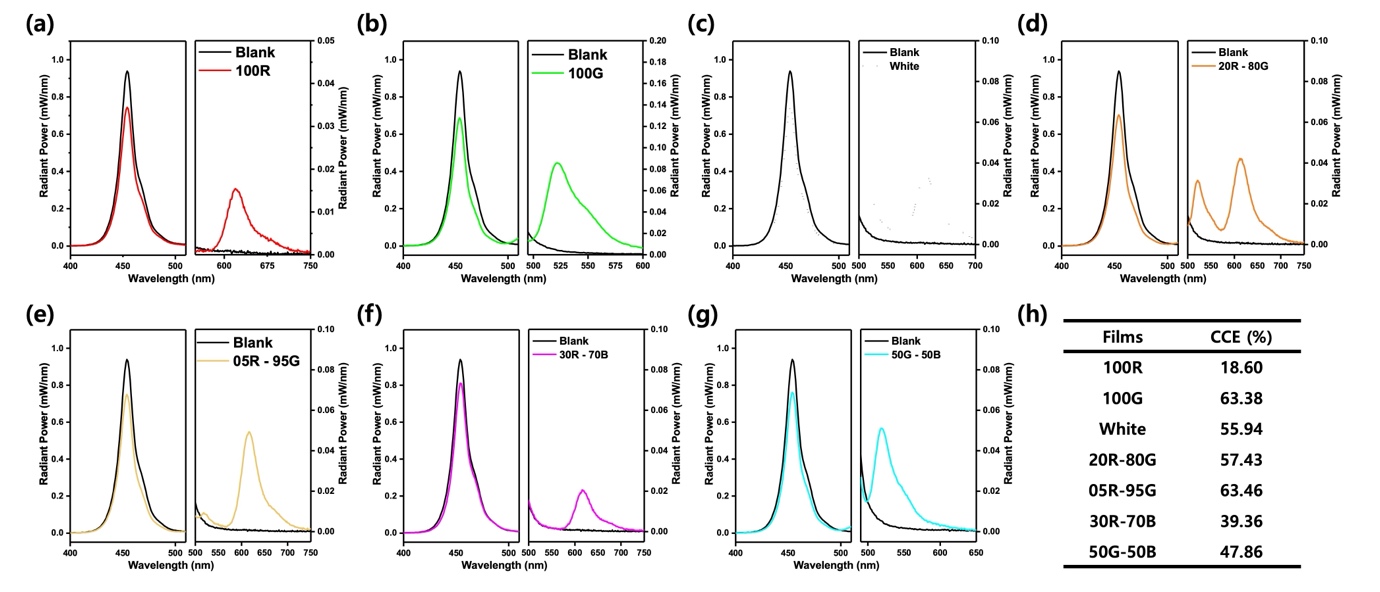


**Figure S11.** Color conversion efficiency of all films.


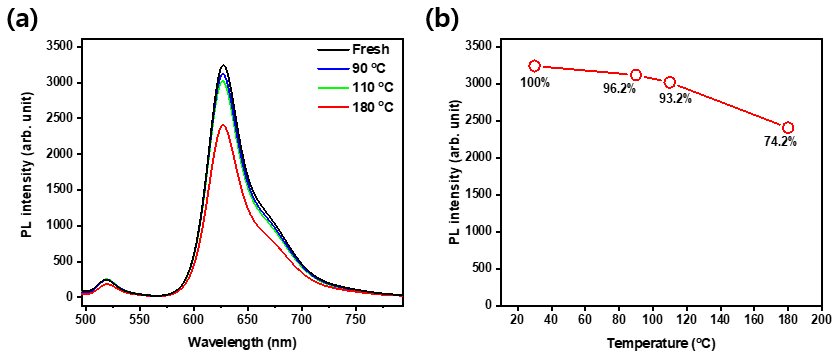


**Figure S12.** (a) Photoluminescence (PL) spectra of CTONDs deposited on paper after thermal exposure at various temperatures (90 °C, 110 °C, 180 °C) for 30 minutes, compared to an untreated (fresh) sample. (b) Relative PL intensity as a function of temperature. The CTONDs retain over 93% of their emission intensity up to 110 °C, and ~74% at 150 °C, demonstrating good thermal stability under short-term heating conditions.

**Table S1.** Summary of DLS parameters, including Z-average diameter, polydispersity index (PI), standard deviation (SD), relative standard deviation (RSD), and the intensity-based contribution of each peak.


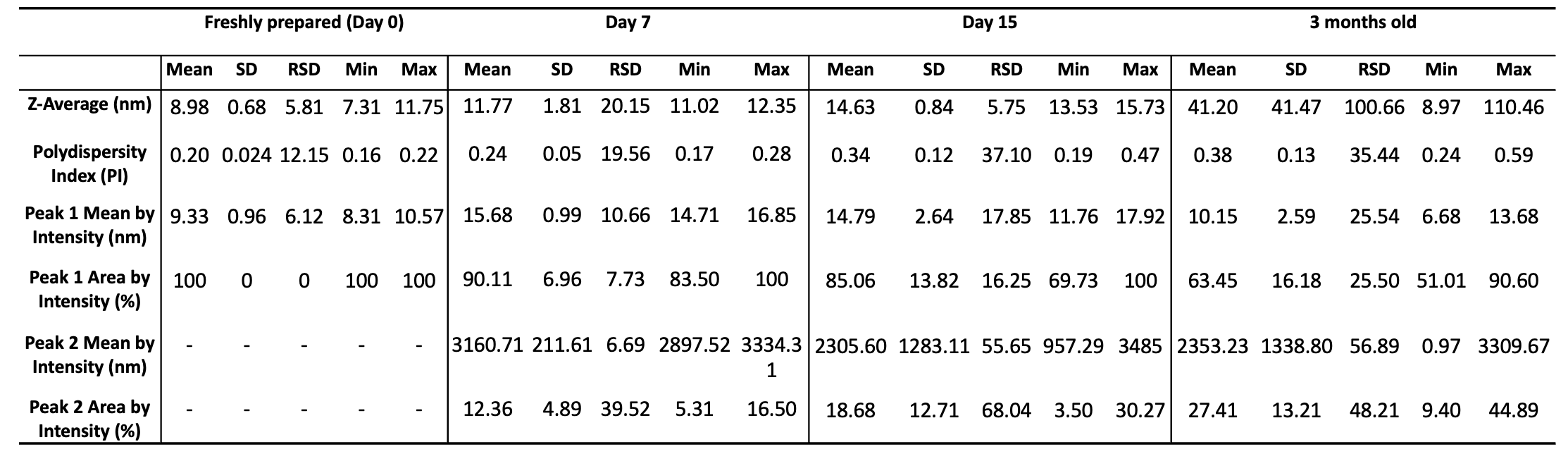


**Table S2.** Comparison between photophysical properties of different nanoparticle system.

| Material Type | PLQY  (%) | CCE  (%) | FWHM  (nm) | Photostability | FRET  (%) | Reference |
| --- | --- | --- | --- | --- | --- | --- |
| CTONDs | 92 (Green)  97 (Blue)  100 (Red) | 63.5 | 35–50 | >4 months | >90 | This work |
| Organic Nano-Dots | 89 | 31 | 35.4 | Stable | - | [1] |
| Carbon dots | 57 | - | - | Stable | ~86 | [2] |
| InP QDs | 61-69 | 21 | 46 | ~30 days | ~82 | [3,4] |
| Perovskite QD | 92 | - | 19 (green),  33 (red) | lifetime >1000h | - | [5] |
| Resin stabilized QD | 85 | 52 | - | Stabilized by resin | - | [6] |

References

[1] Y. Khan, S. Hwang, R. Braveenth, Y. H. Jung, B. Walker, J. H. Kwon, *Nat. Commun.* **2022**, *13*, 1801.

[2] J. Li, S. Zheng, X. Zhao, A. Vomiero, X. Gong, *Nano Energy* **2025**, *134*, 110514.

[3] S.-H. Lee, K.-H. Lee, J.-H. Jo, B. Park, Y. Kwon, H. S. Jang, H. Yang, *Opt. Mater. Express* **2014**, *4*, 1297.

[4] H. V. Demir, S. Nizamoglu, T. Erdem, E. Mutlugun, N. Gaponik, A. Eychmüller, *Nano Today* **2011**, *6*, 632.

[5] J. Lin, S. Chen, W. Ye, Y. Zeng, H. Xiao, T. Pang, Y. Zheng, B. Zhuang, F. Huang, D. Chen, *Adv. Funct. Mater.* **2024**, *34*, 2314795.

[6] B. Karadza, P. Schiettecatte, H. Van Avermaet, L. Mingabudinova, L. Giordano, D. Respekta, Y. H. Deng, I. Nakonechnyi, K. De Nolf, W. Walravens, Y. Meuret, Z. Hens, *Nano Lett.* **2023**, *23*, 5490.
